# Tocilizumab monotherapy uncovered the role of the CCL22/17‐CCR4^+^ Treg axis during remission of crescentic glomerulonephritis

**DOI:** 10.1002/cti2.1203

**Published:** 2020-10-30

**Authors:** Ryota Sakai, Minako Ito, Keiko Yoshimoto, Shunsuke Chikuma, Takahiko Kurasawa, Tsuneo Kondo, Katsuya Suzuki, Tsutomu Takeuchi, Koichi Amano, Akihiko Yoshimura

**Affiliations:** ^1^ Department of Microbiology and Immunology Keio University School of Medicine Tokyo Japan; ^2^ Department of Rheumatology and Clinical Immunology Saitama Medical Center Saitama Medical University Kawagoe Japan; ^3^ Division of Rheumatology Department of Internal Medicine Keio University School of Medicine Tokyo Japan

**Keywords:** ANCA‐associated vasculitis, crescentic glomerulonephritis, CCL22/17–CCR4 axis, IL‐6, M2‐like type macrophages, tocilizumab, Tregs

## Abstract

**Objectives:**

Tocilizumab (TCZ) is a humanised anti‐interleukin (IL)‐6 receptor (IL‐6R) monoclonal antibody that is a promising agent to treat various autoimmune diseases. However, the mechanism of TCZ efficacy is unclear. This study aims to elucidate the relationship between Tregs and IL‐6R blockade in autoimmunity‐mediated renal disease based on a TCZ‐treated cohort of patients with anti‐neutrophil cytoplasmic antibody (ANCA)‐associated vasculitis (AAV) and in an experimental model of crescentic glomerulonephritis (cGN).

**Methods:**

We examined multiple serum levels of cytokines and chemokines and peripheral blood mononuclear cells in patients with AAV who received TCZ monotherapy and achieved drug‐free remission. Moreover, we investigated the mechanistic role of IL‐6R blockade in accelerated cGN model to analyse the local sites of inflammation.

**Results:**

Serum chemokines CCL22 and CCL17, in addition to the CCR4^+^Foxp3^+^ Treg population, increased in patients who demonstrated drug‐free remission after the cessation of TCZ. In the cGN model, IL‐6R blockade ameliorated the disease, elevated CCL22/17 in CD206^+^CD11b^+^CD11c^+^ kidney M2‐like type macrophages, and increased the migration of Tregs into the kidney and regional lymph nodes. The local administration of CCL22 in the kidney facilitated Treg accumulation and reduced glomerular crescent formation.

**Conclusions:**

This study revealed a new mechanism whereby effector Tregs migrate into the inflammatory kidney *via* the CCL22/17–CCR4 axis that is facilitated by M2‐like type macrophages that are induced by IL‐6R blockade.

## Graphical abstract

Our study has revealed a new remission mechanism whereby effector Tregs migrate into the crescentic glomerulonephritis via the CCL22/17–CCR4 axis that is facilitated by M2‐like type macrophages that are induced by IL‐6R blockade.

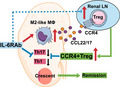

## Introduction

With the introduction of biologics to rheumatoid arthritis, tocilizumab (TCZ), a humanised anti‐interleukin (IL)‐6 receptor (IL‐6R) monoclonal antibody, is a promising agent in various autoimmune diseases, such as large vessel vasculitis (giant cell arteritis and Takayasu arteritis) and adult‐onset Still's disease.^1^, ^2^, ^3^ Moreover, we and others have recently reported that TCZ is an effective treatment in some patients with anti‐neutrophil cytoplasmic antibody (ANCA)‐associated vasculitis (AAV).^4^, ^5^, ^6^, ^7^ Although IL‐6 plays an important role in the pathogenesis of many autoimmune diseases, the mechanism of TCZ efficacy remains unclear.

Tregs are pivotal in peripheral tolerance and tissue homeostasis in the immune system.^8^, ^9^ They have been implicated in the long‐term tolerance of autoimmune diseases, allergic diseases, and the suppression of rejection following organ transplantation; however, the clinical application of Tregs has been limited to date.^10^ Before Tregs can be used clinically, the evidence and mechanisms of Treg‐mediated tolerance *in vivo* must be identified.

In autoimmune kidney diseases, IFNγ‐secreting Th1 cells and IL‐17‐producing Th17 cells accumulate in the kidneys of patients with AAV and glomerulonephritis (GN).^11^, ^12^ Furthermore, for pathogenesis, these cells are critical in an experimental mouse model of crescentic GN (cGN),^12^, ^13^, ^14^, ^15^, ^16^ which is the main symptom of AAV. However, the accumulation and survival of Tregs at sites of local inflammation in the kidney are essential for disease suppression.^11^, ^17^, ^18^, ^19^, ^20^ The roles of chemokine receptors CXCR3 and CCR6 on Tregs have been demonstrated in a murine cGN model.^11^, ^17^ However, the involvement of the chemokine ligand/receptor systems of Tregs in patients with autoimmune diseases in remission after therapy remains unclear.

From our clinical trial, we noticed that certain patients with AAV achieved drug‐free remission for some years after 1 year of TCZ monotherapy. In patients in remission, serum levels of macrophage‐derived chemokine (MDC/CCL22) and thymus and activation‐regulated chemokine (TARC/CCL17), which are ligands of CCR4,^21^ were increased during and after TCZ treatment, accompanied by an increase in the proportion of CCR4^+^ Tregs in the patients' peripheral blood T cells.

To examine the role of the CCL22/17–CCR4^+^ Treg axis in autoimmune renal disease during TCZ treatment, we employed an extensively used mouse model of cGN, as previously established.^11^, ^12^ Our results demonstrated that the CCL22/17–CCR4^+^ Treg axis plays an important role in maintaining tolerance for cGN in both humans and mice.

## Results

### Elevation of CCL22/17 and CCR4^+^ Tregs in patients with AAV with drug‐free remission

**Figure 1:**
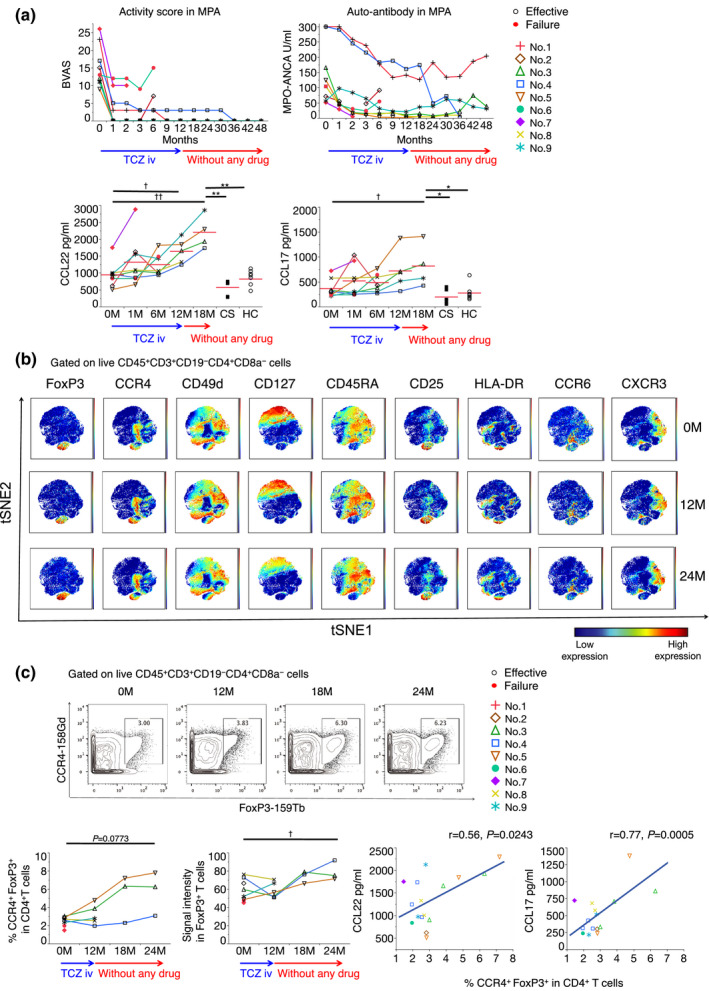
TCZ monotherapy increased serum chemokine (CCL22/17) levels in patients with AAV, even after treatment cessation. Clinical background is shown for each patient with microscopic polyangiitis (MPA) in Supplementary tables 1–3. MPA patients received 8 mg kg^–1^ of intravenous TCZ fortnightly for the first 2 months and monthly for the next 10 months (total 15 courses). (**a**) Changes in clinical disease activity were observed in MPA (BVAS and MPO‐ANCA). Symbols represent individual data points, and each line shows the change in individual cases. The open symbol represents responders, whereas the red filled symbol represents non‐responders. BVAS, Birmingham Vasculitis Activity Score; MPO‐ANCA, myeloperoxidase–anti‐neutrophil cytoplasmic antibody. Serum levels of CCL22 (left) and CCL17 (right) were measured during and after treatments at 0, 1, 6 and 12 months during TCZ monotherapy and at 18 months in untreated patients, MPA patients in remission with only corticosteroid treatments, and healthy controls. (**b**) Representative viSNE analysis as part of mass cytometry performed on eight patients and five healthy controls individuals using their blood mononuclear cells gated on live CD45^+^CD3^+^CD4^+^ cells. (**c**) Representative two‐dimensional analysis of CCR4^+^ and FoxP3^+^ cells from mass cytometry analyses of blood mononuclear cells gated on live CD4^+^CD3^+^CD45^+^ T cells. Among five patients who remained in remission for 1 year, three showed proportions of CCR4^+^ Treg and signal intensity of Foxp3^+^ cells gated on live CD4^+^CD3^+^CD45^+^ T cells; correlation diagram of CCL22/17 and CCR4^+^ Treg among all patients at any timepoint. Patient 1 had no data at any time point, and patient 9 had no data at 18 and 24 months; red filled symbols represent non‐responders. ^*,†^ *P* < 0.05 and ^**,††^ *P* < 0.01 (^*^Wilcoxon rank‐sum test and ^†^Wilcoxon signed‐rank test).

Based on our previous study,^6^ we analysed a total of nine patients with AAV, (all with microscopic polyangiitis, MPA, which is highly prevalent in Japan^22^) who received 12 months of TCZ monotherapy without corticosteroids or immunosuppressants, as a first induction treatment soon after diagnosis (Supplementary table 1). Assessments using Birmingham Vasculitis Activity Score and myeloperoxidase (MPO)‐ANCA assays showed that seven of the nine patients responded to TCZ treatment, and no recurrence was observed in six of these seven patients after TCZ treatment cessation (Figure 1a). Moreover, five patients achieved remission or at least low‐disease activity for 2 years after TCZ treatment.

Note that the serum levels of CCL22/17 in the patients with AAV increased during treatment and remained increased thereafter, which was not seen in AAV patients treated with low‐dose corticosteroids on maintenance therapy or in healthy controls (Figure 1b, Supplementary tables 2 and 3). Despite the involvement of CXCR3^+^ and CCR6^+^ Tregs in the renal biopsy sample in patients with AAV,^11^ the levels of CXCL10 (a CXCR3 ligand) and CCL20 (the CCR6 ligand) decreased or remained unchanged (Supplementary figure 1). These data indicate that CCL22/17 are unique chemokines that could be involved in the remission states of AAV.

Next, we examined changes in the subsets of peripheral blood mononuclear cells (PBMCs) using mass cytometry (Supplementary figure 2a and b). The proportion of effector Tregs (eTregs: FoxP3^+^CD49d^−^CD127^low^CD25^high^HLADR^+^CD45RA^−^) increased in all three of the confirmed samples of patients at one‐year remission following the cessation of TCZ treatment. Moreover, CCR4^+^Foxp3^+^ Tregs and signal intensity of FoxP3^+^ were increased (Figure 1c and d). Furthermore, the serum levels of CCL22/17 and the percentage of CCR4^+^Foxp3^+^ Tregs were positively correlated (Figure 1d). In myeloid cells, we noticed increased CD11b^+^CD11c^+^ cells, particularly those of CD163^+^, a human M2 macrophage marker in monocyte/DC/macrophages, after TCZ treatment (Supplementary figure 2c). These data suggest that TCZ changes the immune environment in patients with AAV, resulting in drug‐free remission.

### IL‐6 blockade enhanced Treg accumulation and M2‐like type macrophage activation with an increase in CCL22/17 in the kidney

To investigate the effect of the anti‐IL‐6R antibody *in vivo*, we used a well‐characterised T‐cell**‐**mediated mouse model of cGN (nephrotoxic nephritis), as in previous studies^11^, ^12^ (Supplementary figure 3a and b). A previous study using the cGN model noted that pathogenic Th17 levels in the kidney peaked at day 10 (early phase) after induction, corresponding with disease activity.^12^, ^16^ Increases in pathogenic Th1 and Treg cells plateaued on day 21 (late phase), whereas Th17 cells plateaued earlier, and Tregs were maintained at 30–40% for at least 3 months. Note that Treg depletion in this model exacerbates cGN^23^, ^24^; therefore, Tregs have an important role in maintaining disease activity by suppressing Th1 and Th17 cells in this model.

**Figure 2:**
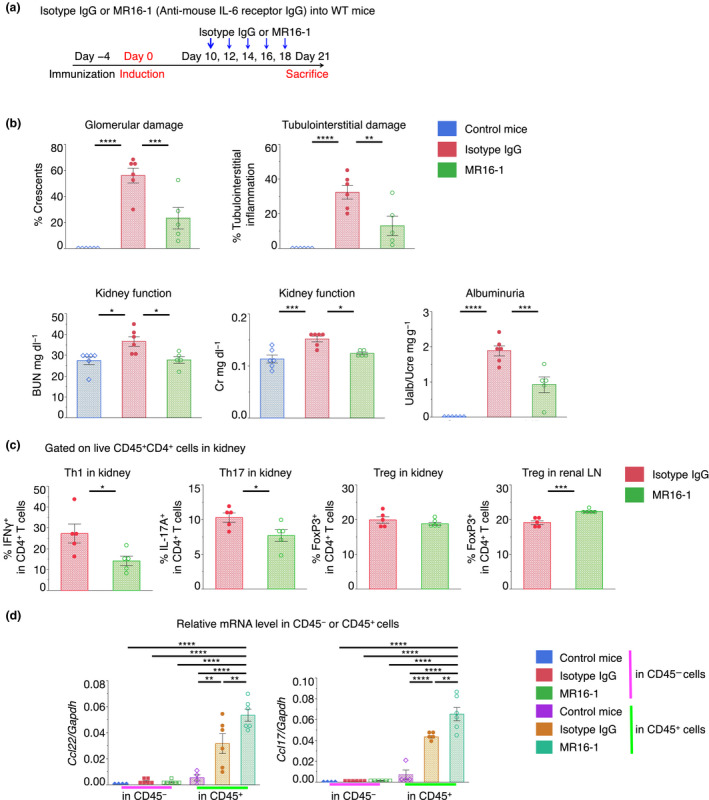
Anti‐mouse IL‐6R antibody treatment ameliorated crescentic glomerulonephritis (cGN) in WT mice. (**a**) Schematic for MR16‐1 treatment regimen; intravenous infusions of MR16‐1 at an initial dose of 2 mg per mouse and 0.5 mg per mouse thereafter; (**b**) glomerular crescent formation and interstitial inflammation were evaluated according to renal pathological findings. Kidney function was assessed by measuring serum blood urea nitrogen (BUN) levels and determining ratios of urine albumin (UAlb)/urine creatinine (UCre) (n = 5 or 6). (**c**) Proportions of IFNγ^+^, IL‐17A^+^, and FoxP3^+^ cells per FVD^−^CD45^+^CD4^+^ cell in the kidney and FoxP3^+^ cells per FVD^−^CD45^+^CD4^+^ cell in the renal LN (n = 5); (**d**) CCL22/17 mRNA expression levels were normalised to those of GAPDH in CD45^+^ and CD45^−^ cells that were isolated from kidneys (n = 4 or 6). Symbols represent individual data points for mice in representative independent experiments of three. Horizontal lines indicate mean ± SEM; ^*^ *P* < 0.05, ^**^ *P* < 0.01, ^***^ *P* < 0.001 and ^****^ *P* < 0.0001 (Tukey–Kramer HSD test).

The effect of IL‐6 blockade on this murine cGN model remains controversial.^20^, ^25^, ^26^ In our model, an *Il‐6* ^−/−^ background reduced disease severity (Supplementary figure 4a and b). Furthermore, anti‐IL‐6R antibody (MR16‐1) treatment at the early phase ameliorated pathological results (Supplementary figure 5a and b). Moreover, MR16‐1 on day 10 reduced disease severity, suggesting that IL‐6‐blockade affects both early and late phases, even after the disease severity peaked (Figure 2a and b). MR16‐1 treatment increased Tregs in draining (renal) lymph nodes on day 21 (Figure 2c). Moreover, the expression of CCL22/17 in CD45^+^ cells was higher than that in CD45^−^ cells of the kidney (Figure 2d).

**Figure 3:**
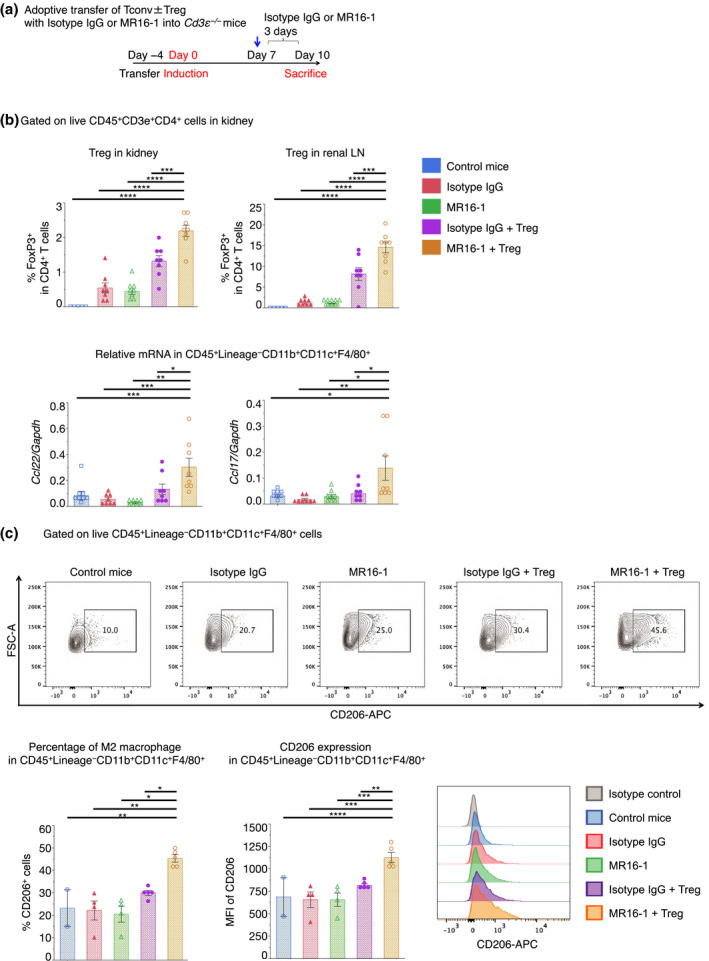
MR16‐1 increased Treg migration into the kidney and induced CCL22/17 expression in macrophages following adoptive transfer in *Cd3ε^−/−^* mice. (**a**) Schematic showing methods for inducing cGN in *Cd3ε^−/−^* mice by adoptive transfer of CD4^+^FoxP3^–^hCD2^+^ (Treg: 7.5 × 10^5^) or CD4^+^FoxP3^–^hCD2^−^ (Tconv: 3.0 × 10^6^) cells and MR16‐1 treatments (intravenous infusion of 1 mg per mouse at day 7). (**b**) Proportions of FoxP3^+^ cells per FVD^−^CD45^+^CD3^+^CD4^+^ cell in kidney, and renal LN (n = 5 or 8). CCL22/17 mRNA expression levels were normalised to those of GAPDH in FVD^−^CD45^+^, lineage negative, and CD11b^+^CD11c^+^F4/80^+^ cells after isolation from kidneys (n = 8). (**d**) Representative dot plots of flow cytometry (FCM) analysis gated on FVD^−^CD45^+^, CD4^−^CD19^−^CD49b^−^Ly6G^−^ (lineage negative), and CD11b^+^CD11c^+^F4/80^+^ cells in kidneys; proportions of CD206^+^ cells per FVD^−^CD45^+^, lineage negative, and CD11b^+^CD11c^+^F4/80^+^ cell in the kidney (left); quantification of mean fluorescence intensities (MFI) of CD206 in CD45^+^, lineage negative, and CD11b^+^CD11c^+^F4/80^+^ cells in the kidney (right; n = 2, 4 or 5). Symbols represent individual data points for mice that were representative of three independent experiments, and the horizontal lines indicate mean ± SEM; ^*^ *P* < 0.05, ^**^ *P* < 0.01, ^***^ *P* < 0.001, ^****^ *P* < 0.0001 (Tukey–Kramer HSD test).

We suspected that we were unable to detect any increase of Tregs in the kidney because of the considerable improvement in the disease by MR16‐1. Therefore, we established a method of Treg transfer into *Cd3ε^−/−^* mice lacking T cells before disease induction (Figure 3a). Note that conventional T cells (CD4^+^ FoxP3^–^hCD2^−^: Tconv) were transferred on day −4 with or without Tregs (CD4^+^ FoxP3^–^hCD2^+^) into *Cd3ε^−/−^* mice. In this setting, MR16‐1 significantly increased the accumulation of Tregs into the kidney, and especially, the draining lymph nodes (Figure 3b and Supplementary figure 6).

Next, we examined the expression of CCL22/17 in CD45^+^ cells. We sorted all CD45^+^ cells to detect the expression of CCL22/17 and finally observed that CCL22/17 was mostly expressed in CD11b^+^CD11c^+^F4/80^+^ cells of the kidney (Figure 3c). Note that various types of macrophages infiltrate into the kidney after renal diseases,^27^ that CCL22/17 are expressed in CD206^+^ M2‐like type macrophages,^28^ and that the transfer of CD206^+^ M2 macrophages ameliorates pathology in the cGN model.^29^ Consistently, we reported that Treg transfer and MR16‐1 treatment increased the CD206^+^CD11b^+^CD11c^+^F4/80^+^ macrophage fraction (Figure 3d and Supplementary figure 3c). These data suggest that the blockade of IL‐6R promotes the polarisation of macrophages into an M2‐like type, thereby increasing the expression of CCL22/17.

### Local administration of CCL22 in the kidney facilitated Treg migration and reduced glomerular crescent formation

**Figure 4:**
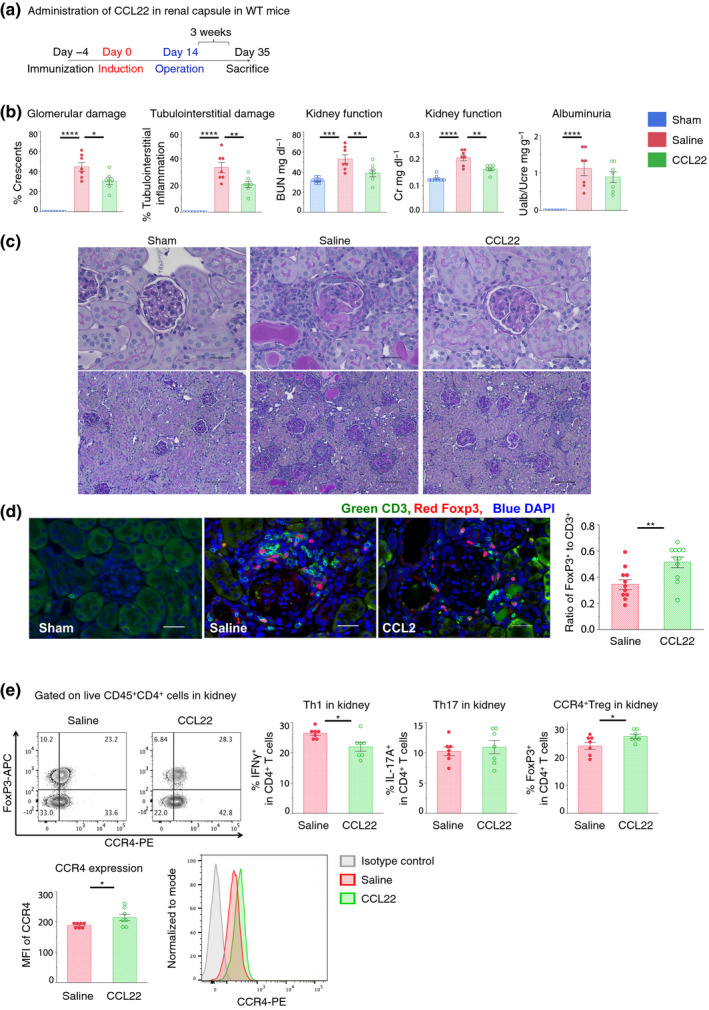
Local administration of CCL22 facilitated Treg migration and reduced glomerulonephritis symptoms. (**a**) Schematic of the CCL22 treatment regimen; 4 μg per mouse and 2 μg per kidney; procedure in detail was described in Supplementary table 4. (**b**) Glomerular crescent formation and interstitial inflammation were evaluated in renal pathological observations. Kidney function was assessed by measuring serum blood urea nitrogen (BUN) and creatinine levels, and according to ratios of urine albumin (UAlb)/urine creatinine (UCre) (n = 7 or 8). Symbols represent individual data points from mice that were representative of two independent experiments. Horizontal lines indicate mean ± SEM; ^*^ *P* < 0.05, ^**^ *P* < 0.01, ^***^ *P* < 0.001, ^****^ *P* < 0.0001 (Tukey–Kramer HSD test). (**c**) Representative photomicrographs were obtained at 600× (upper) and 100× (lower) magnification using a light microscope and depict crescentic glomeruli and interstitial inflammation on PAS staining; scale bar, 30 μm (upper) and 200 μm (lower). (**d**) Photomicrographs were obtained at 600 × magnification using indirect immunofluorescence to depict crescentic glomeruli; green, CD3; red, FoxP3; blue, DAPI; scale bar, 30 μm. Symbols represent a glomerulus from tissue sections using immunofluorescence that were representative of two independent experiments. Horizontal lines indicate mean ± SEM; ^*^ *P* < 0.05 (Student's *t*‐test) (**e**) Representative dot plots from FCM analyses gated on FVD^−^CD45^+^CD4^+^ cells that were isolated from kidneys. Proportions of IFNγ^+^, IL‐17A^+^ and CCR4^+^FoxP3^+^ cells per FVD^−^CD45^+^CD4^+^ cell in the kidney (n = 7 or 8). Representative histograms of CCR4 expression and quantification of mean fluorescence intensities (MFI); symbols represent individual data points from mice that were representative of two independent experiments. Horizontal lines indicate mean ± SEM; ^*^ *P* < 0.05 (Student's *t*‐test).

We previously reported that Tregs ameliorated renal dysfunction in a CCR4‐dependent manner.^30^ Thus, we administered recombinant CCL22 locally under renal capsules using Medgel^®^, which allows the sustained release of physiologically active substances *in vivo*. In the late phase, this treatment improved renal function and decreased the formation of crescentic glomeruli (Figures 4a–c). Immunohistochemical staining of kidney sections revealed an increased ratio of Treg cells to CD3^+^ cells, even around the non‐crescentic glomeruli (Figure 4d). Moreover, we observed increased ratios of CCR4^+^ Tregs and expression levels of CCR4 in Tregs (Figure 4e). The administration of CCL17 rather than CCL22 under the renal capsules did not improve renal function or change the CCR4^+^ Treg ratios (Supplementary figure 7a–c). The expression level of *Ccl17* mRNA in the kidney was higher than that of *Ccl22* mRNA during the chronic phase (Supplementary figure 7d). A previous study demonstrated that the receptor CCR4 binds with threefold lower affinity to CCL17 than to CCL22.^31^ In fact, CCL22 had a higher migration ability than CCL17 (Supplementary figure 7e), suggesting that CCL22, rather than CCL17, is important in suppressing disease severity. To summarise, the data demonstrate that CCL22 facilitates CCR4^+^ Treg infiltration into kidney tissues and contributes more toward improving renal function, indicating that CCL22 has a therapeutic potential.

## Discussion

**Figure 5:**
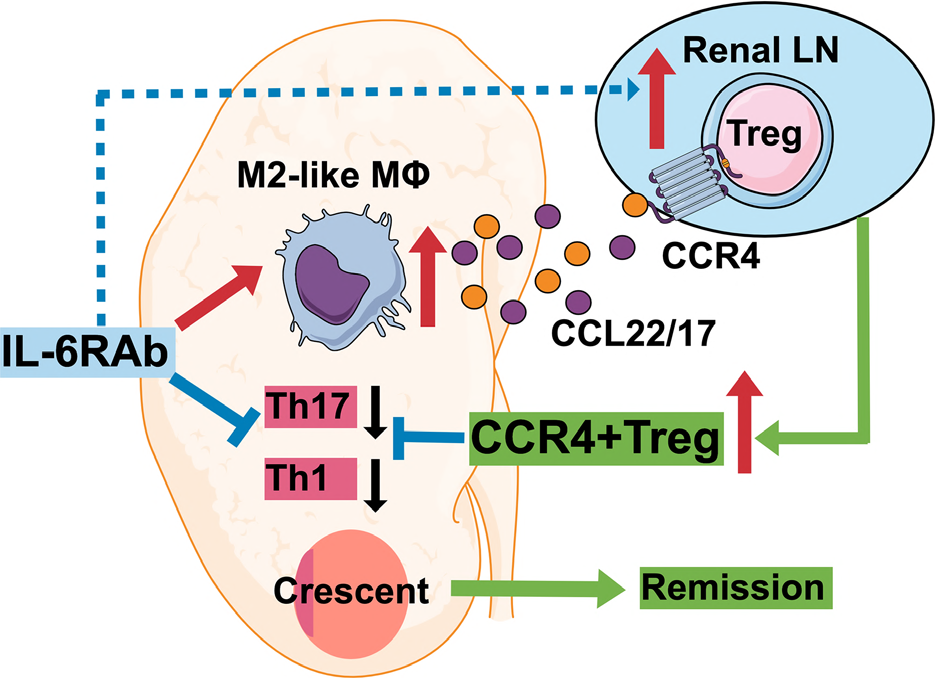
In this study, we revealed a new remission mechanism whereby not only suppressing Th1 and Th17 cells but also effector Tregs migrate into the crescentic glomerulonephritis *via* the CCL22/17–CCR4 axis that is facilitated by M2‐like type macrophages that are induced by IL‐6R blockade.

Our clinical observations in AAV patients treated with TCZ monotherapy increased the possibility of the involvement of the CCL22/17–CCR4^+^Treg axis in the tolerance of cGN. Using a cGN mouse model, we demonstrated that IL‐6R blockade increased CCL22/17 expression by promoting M2‐type macrophage development and facilitated CCR4^+^ Treg migration into inflammation sites (Figure 5). Although the importance of CCL22/17–CCR4 axis has been reported in various mouse models,^32^, ^33^, ^34^, ^35^, ^36^ to the best of our knowledge, our study is the first to clarify that the effect of IL‐6 signalling blockade reinforces this axis in cGN.

cGN is the most severe pathological kidney disease and results in end‐stage renal failure. It is associated with high morbidity, mortality and overwhelming medical expenses.^37^ Although different pathogenic mechanisms, such as systemic lupus erythematosus and IgA nephropathy, can lead to the development of cGN, AAV is the most common cause of cGN. This disease is pathologically characterised by the formation of glomerular crescents with pauci‐immune deposition. Several studies have used various animal models of AAV; however, all of them have debatable inconsistencies with the human disease.^38^ In this study, the murine cGN model used may not be perfectly parallel to the pathology of AAV; however, it has been extensively used to understand immune‐mediated forms of GN, and previous studies have demonstrated its suitability for analysing the role of T‐cell‐mediated immune systems in AAV with nephropathy.^11^, ^12^ Although there is a disconnect between our results from patients with AAV and the results from our mouse model and there are limitations due to the small sample size, we demonstrated an important role for CCL22/17–CCR4^+^ Tregs induced by IL‐6R blockade in the mouse model, as observed in the human study during remission.

Although the expression level of CCL22/17 increased during MR16‐1 administration to wild‐type (WT) mice, no change was observed in the proportion of endogenous Tregs in the kidney, except for an increased proportion of Tregs in the regional lymph node. Furthermore, we assumed that the *in vivo* detection of the trajectories of Treg was not precise. The IL‐6R blockade had a direct effect on the inhibition of inflammatory environments, such as decreasing Th1/17. After the inflammation had resolved, IL‐6R blockade might have effectively accelerated Treg adaptation into the peripheral tissue *via* the CCL22/17–CCR4 axis. Assuming this is the case in humans, the CCL22/17–CCR4 axis would not effectively work as it is suppressed by conventional corticosteroid treatment. This study does not indicate whether TCZ by itself is clinically safe and effective for AAV; however, from the viewpoint of clinical significance, our results may lead to a crucial consideration for the short‐term use of corticosteroids in daily clinical practice.

Unlike our study, some studies show that the IL‐6 signalling pathway protects against cGN in mice on macrophages and CD4^+^ T cells at the early disease phase.^20^, ^26^ The reason for the discrepancy between these studies and ours is unclear at present. One of these studies used a low dose of MR16‐1, which may have resulted in insufficient blockage of IL‐6R.^26^ Indeed, in our study, MR16‐1 induced the expression of IL‐6 on mononuclear cells, which may have caused a paradoxical effect on cGN by insufficient blockade (data not shown). Moreover, in our human study, the serum IL‐6 levels at 4 weeks significantly increased from the baseline, as in a previous study.^39^ Thus, IL‐6 signalling and the CCL22/17–CCR4 axis exert different effects depending on the difference in the types of disease model, phase and specific cells.

Although the specific mechanism by which the blockade of IL‐6R elevates CCL22/17 remains unclear, it might be a reason why IL‐4 expression on T cells is caused by IL‐6 blockade suppression (Supplementary figure 5c and d). IL‐4 ameliorates cGN in Wistar Kyoto rats.^40^ Furthermore, IL‐4 activates M2 macrophage‐related genes and promotes the production of CCL22/17,^28^ and IL‐6 influences the balance between M1 and M2 macrophages.^41^, ^42^ By blocking IL‐6, IL‐4 expression and subsequent M2 macrophage activation might increase the expression of CCL22/17, resulting in the migration of CCR4^+^ Tregs to the sites of inflammation. CCL22 and CCL17 are produced by cancer cells and are known to induce immune evasion in cancer. Thus, similar to immune evasion, increase of CCL22/17 might play an important role in promoting Treg migration, resulting in immune tolerance during and after TCZ treatment.

Analyses of human regulatory T cells revealed that eTregs (CD25^high^CD45RA^−^CD4^+^) highly express CCR4 but not CD49d, IL‐17A and IFNγ, while CCR4 expression was reduced in Treg ‘Fraction III’ that produces effector cytokines, despite expressing FoxP3.^43^, ^44^ These data indicate that CCR4 is crucial for the migration of Tregs into the local sites of inflammation and for peripheral tolerance in humans. In previous studies of rheumatoid arthritis and giant cell arteritis, TCZ treatments increased Treg proportions.^45^, ^46^ However, little is known of how Tregs migrate and increase in the tissue. Our data suggest that the CCL22/17–CCR4^+^ Treg axis may be involved in long‐term disease tolerance in patients with autoimmunity‐mediated renal disease.

The difference between CCL22 and CCL17 is uncertain. Both chemokines activate the chemokine receptor CCR4 and are structurally related; however, their mode of action is thought to be different.^47^ In our study, CCL22 suppressed the severity of cGN as determined by kidney function and pathological findings, whereas CCL17 did not. Moreover, CCL22 had a greater ability to migrate than CCL17. The expression level of *Ccl17* mRNA in the kidney was higher than that of *Ccl22* mRNA in the chronic phase, indicating that CCL17 may require a higher threshold dose than that of CCL22 to suppress cGN activity. Thus, additional studies are required to reveal the mechanisms of secretion and action of CCL22 and CCL17 in mice and humans.

In conclusion, our studies have revealed a new mechanism whereby effector Tregs migrate into the inflammatory kidney via the CCL22/17–CCR4 axis that is facilitated by M2‐like type macrophages that are induced by IL‐6R blockade.

## Methods

### Human participants

Patients with microscopic polyangiitis (MPA) and healthy control (HC) subjects were recruited for the study (Supplementary tables 1–3). Eligible patients were aged 20–80 years and diagnosed with MPA according to the Watts' classification. The treatment protocol was as shown previously.^6^ All human subjects provided written informed consent for the study and provided serum and PBMCs.

### Mice

C57BL/6J mice and *Il‐6^−/−^*, *Foxp3^–^hCD2*, and *Cd3ε^−/−^* mice were purchased from Tokyo Laboratory Animals Science Co., Ltd or Jackson Laboratory. All mice were on a C57BL/6 genetic background. Male mice aged 8–12 weeks and weighing 20–30 g were used under co‐housing conditions in specific pathogen‐free facilities.

### Induction of experimental cGN model

The cGN model^48^ was accelerated by sensitising mice to normal rabbit globulin (0.25 mg) with complete Freund's adjuvant and by adding *Mycobacterium tuberculosis* (H37Rv) 0.5 mg per mouse (Difco Laboratories). Four days after immunising, rabbit anti‐mouse anti‐glomerular basement membrane (GBM) antibodies with lipopolysaccharide (Sigma‐Aldrich; *Escherichia coli* O26:B6 L8274) 25 ng per 1 mg GBM antibodies were injected as previously reported.^49^, ^50^ Renal function was measured by Oriental Yeast Co., Ltd.

### Histologic assessment

The renal cortex was fixed in paraformaldehyde overnight, and 4 μm‐sized tissue sections were cut and stained with periodic acid–Schiff (PAS). A glomerulus was considered to exhibit crescent formation when two or more layers of cells were observed in Bowman's space. A minimum of 100 glomeruli was assessed to determine the crescent score. For evaluating tubulointerstitial inflammation, 10 sites on the cortical area were randomly selected, and the average ratio of leukocytic infiltration, tubular dilation, tubular atrophy and cast formation were determined at a magnification of 200× per previous studies^14^, ^18^, ^24^ using a BZ‐X700 fluorescence microscope (Keyence). For assessing Treg migration to the glomeruli, at least 10 glomeruli were counted to determine the number of FoxP3^+^ cells and CD3^+^ cells from tissue sections using immunofluorescence.

### Antibodies, reagents and *in vivo* experiments

All products and *in vivo* experiments are described in Supplementary table 4.

For mass cytometry analyses, cryopreserved PBMCs were processed as per the instructions for Cytobank analyses (Cytobank, Inc.), which were performed at St. Luke's MBL Corporation.

For flow cytometry, kidney cells were obtained from mice with saline perfusion, digested and filtered at 70 μm, and they were isolated using an autoMACS with anti CD45 microbeads (Miltenyi Biotec) after Fc blocking by positive selection (Possel). They were acquired with a FACSCanto II (BD Biosciences) and analysed using FlowJo 9.9.3 (FlowJo, LLC).

For adoptive transfer, we isolated CD4^+^ cells from the spleen of mice using an autoMACS Pro Separator with a CD4 isolation kit by negative selection (Depletes) and then sorted by a Sony SH800 cell sorter (Sony Biotechnology).

For *in vivo* transplant experiments, recombinant CCL22 or CCL17 (PeproTech) was dissolved in Medgel^®^ (Medgel Pvt., Ltd) and surgically transplanted under both kidney capsules (see Supplementary table 4).

### *In vitro* cell migration assay

Treg cells (4.0 × 10^5^ per well) were isolated from the spleen and lymph nodes of *Foxp3‐hCD2* mice after disease induction using an autoMACS Pro Separator with a CD4 isolation kit by negative selection (Depletes) and then positive selection (Possel_2), achieved by anti‐phycoerythrin (PE) beads in the presence of PE‐hCD2 (TS1/8). Then, cells were sorted using PE‐hCD2, allophycocyanin (APC)‐CD4 (RM4‐5), and brilliant violet (BV) 421‐CD45 (30‐F11) with the Sony SH800 cell sorter. Moreover, *in vitro* cell migration was assayed using a CytoSelect 96‐well cell migration assay kit (5 μm, CBA‐105) (Cell Biolabs) as per the manufacturer's protocol. Migration was stimulated by CCL22/17 (50 ng mL^−1^) in the lower chamber; moreover, no serum was added to the upper chamber. The incubation period was 4–5 h, and the cell lysis buffer was transferred to a 96‐well plate. Relative fluorescence units were then measured by a plate reader at 480 nm/520 nm.

### Quantitative PCR (qPCR)

mRNA was extracted from renal tissues or cells using a FACSAria (BD Biosciences) or autoMACS using RNAiso (TaKaRa Bio Inc.) or ReliaPrep RNA Cell Miniprep System (Promega), respectively. Total RNA was reverse transcribed to cDNA using a high‐capacity cDNA reverse transcription kit (Applied Biosystems). qPCR was then performed on the cDNA samples using EvaGreen (Bio‐Rad). The relative quantification value is expressed as 2^−ΔCt^, in which ΔCt is the difference between the mean Ct value of triplicate measurements and the endogenous GAPDH control.

### Statistics

Statistical significance was determined by the Tukey–Kramer honestly significant difference (HSD) test or Steel–Dwass test to analyse the differences among ≥ 3 groups using the unpaired two‐sided Student's *t*‐test or two‐sided Wilcoxon rank‐sum test between two groups and using the Wilcoxon signed‐rank test between two paired groups. Pearson's correlation coefficient was used to calculate correlation analysis. *P* < 0.05 was considered a significant difference, and all analyses were performed using JMP 12 (SAS Institute Inc.).

### Study approval

This study was approved by the institutional review board of Saitama Medical University (837, 877, 916, and 1109) and Keio University (20160055 and 20160134). The clinical trials were registered with the UMIN clinical trials registry (000011242). This study was performed according to the guidelines stipulated by the World Medical Declaration of Helsinki and the Ethical Guidelines for Clinical Research in Japan, revised in 2008. Animal experiments were performed in strict accordance with the recommendations in the Guidelines for Proper Conduct of Animal Experiments of the Science Council of Japan. All experiments were approved by the Animal Research Committee and Ethics Committee of Keio University.

## Conflict of Interest

TKu has received speaking fees from Asahikasei Pharma Corp.; Astellas Pharma Inc.; Chugai Pharmaceutical Co., Ltd; Eisai Co., Ltd; Eli Lilly Japan K.K.; Mitsubishi Tanabe Pharma Corp.; Sanofi S.A.; and Teijin Pharma Ltd. KS has received research grants from AbbVie GK.; Bristol‐Myers Squibb K.K.; Chugai Pharmaceutical Co., Ltd; Daiichi Sankyo Co., Ltd; Eisai Co., Ltd; Kissei Pharmaceutical Co., Ltd; Ono Pharmaceutical Co., Ltd; Fuji Film; Mitsubishi Tanabe Pharma Corp.; Pfizer Japan Inc.; and Takeda Pharmaceutical Co., Ltd. He has also received personal fees and non‐financial support from AbbVie GK.; Astellas Pharma Inc.; Bristol‐Myers Squibb K.K.; Chugai Pharmaceutical Co., Ltd; Daiichi Sankyo Co., Ltd; Eisai Co., Ltd; Eli Lilly Japan K.K.; Kissei Pharmaceutical Co., Ltd; Fujifilm Corp.; Janssen Pharmaceutical K.K.; Mitsubishi Tanabe Pharma Corp.; Shionogi & Co., Ltd; Pfizer Japan Inc.; Takeda Pharmaceutical Co., Ltd; and UCB Japan Co., Ltd. TT has received research grants from Astellas Pharma Inc.; Chugai Pharmaceutical Co., Ltd; Daiichi Sankyo Co., Ltd; Takeda Pharmaceutical Co., Ltd; AbbVie GK.; Asahikasei Pharma Corp.; Mitsubishi Tanabe Pharma Corp.; Pfizer Japan Inc.; Eisai Co., Ltd; Ayumi Pharmaceutical Co., Ltd; Nipponkayaku Co., Ltd; and Novartis Pharma K.K. TT has also received speaking fees from AbbVie GK.; Bristol‐Myers Squibb K.K.; Chugai Pharmaceutical Co., Ltd; Mitsubishi Tanabe Pharma Corp.; Pfizer Japan Inc.; Astellas Pharma Inc.; Daiichi Sankyo Co., Ltd; Eisai Co., Ltd; Sanofi S.A.; Teijin Pharma Ltd; Takeda Pharmaceutical Co., Ltd; Novartis Pharma K.K.; consultant fees from Astra Zeneca K.K.; Eli Lilly Japan K.K.; Novartis Pharma K.K.; Mitsubishi Tanabe Pharma Corp.; AbbVie GK.; Nipponkayaku Co., Ltd; Janssen Pharmaceutical K.K.; Astellas Pharma; Taiho Pharmaceutical Co., Ltd; Chugai Pharmaceutical Co., Ltd; Taisho Toyama Pharmaceutical Co., Ltd; GlaxoSmithKline K.K.; and UCB Japan Co., Ltd. KA has received research grants from Asahikasei Pharma Corp. and Chugai Pharmaceutical Co. Ltd, and he has received speaking fees from Chugai Pharmaceutical Co. Ltd, Eli Lilly Japan K.K.; Mitsubishi Tanabe Pharmaceutical Corp.; and Pfizer Japan Inc. The other authors have declared that no conflict of interest exists.

## Declaration of Interests

The authors declare that they have no known competing financial interests or personal relationships that could have appeared to influence the work reported in this paper.

## Author Contributions

**Ryota Sakai:** Conceptualization; data curation; formal analysis; investigation; methodology; writing – original draft; writing – review and editing. **Minako Ito:** Data curation; formal analysis. **Keiko Yoshimoto:** Data curation; methodology. **Shunsuke Chikuma:** Data curation; methodology; writing – review and editing. **Takahiko Kurasawa:** Project administration. **Tsuneo Kondo:** Project administration. **Katsuya Suzuki:** Investigation; methodology. **Tsutomu Takeuchi:** Project administration. **Koichi Amano:** Conceptualization; funding acquisition; project administration; supervision. **Akihiko Yoshimura:** Conceptualization; funding acquisition; methodology; project administration; supervision; writing – review and editing.

## Funding information

This study was supported by JSPS KAKENHI (S) JP17H06175, Challenging Research (P) JP18H05376, and AMED‐CREST JP19gm1110009 to AY and Keio University Doctorate Student Grant‐in‐Aid Program to RS. This study was also supported by the Takeda Science Foundation, the Uehara Memorial Foundation, the Mochida Memorial Foundation for Medical and Pharmaceutical Research, Bristol‐Myers Squibb Research grant, the Kanae Foundation, and the SENSHIN Medical Research Foundation.

## Data Availability

The data that support the findings of this study are available from the corresponding author upon reasonable request.
