# Supplementary material for: Tocilizumab monotherapy uncovered the role of the CCL22/17‐CCR4+ Treg axis during remission of crescentic glomerulonephritis
Source: Clin Transl Immunology. 2020 Oct 30;9(11):e1203. doi: 10.1002/cti2.1203 (PMC7596393; doi:10.1002/cti2.1203)
Supplement: Supplementary file 1 [file CTI2-9-e1203-s001.docx]

**SUPPLEMENTARY INFORMATION**

For Sakai et al. **Tocilizumab monotherapy uncovered the role of the CCL22/17-CCR4^+^ Treg axis during remission of crescentic glomerulonephritis**

**Supplementary figure 1. Serum levels of chemokines and cytokines**

**Supplementary figure 2. Gating strategy of mass cytometry and viSNE analysis**

**Supplementary figure 3. Induction of experimental cGN model, representative gating strategy, and dot plots of flow cytometry analysis**

**Supplementary figure 4. Analysis of *Il-6***^−/−^ **mice**

**Supplementary figure 5. Analysis of MR16-1 treatment at the early phase**

**Supplementary figure 6. Representative dot plots of flow cytometry analysis in *Cd3ε^−/−^* mice**

**Supplementary figure 7. CCL17 had a lower migration ability than CCL22**

**Supplementary table 1. Baseline clinical characteristics of patients with MPA in TCZ monotherapy**

**Supplementary table 2. Clinical characteristics of patients with MPA in corticosteroids at a single time of blood sampling**

**Supplementary table 3. Clinical characteristics of healthy control individuals**

**Supplementary table 4. List of products and *in vivo* experimental procedure**

**Supplementary FigureS and Figure CAPTIONS**

**Supplementary figure 1. Serum levels of chemokines and cytokines.**

Serum levels of various cytokines and chemokines in MPA patients at 0 M and 12 M, MPA with corticosteroids (CS), or healthy controls (HC). The black circle represents responders, whereas the red circle represents non-responders. Symbols represent individual data points. Box plots indicate median and interquartile range; ^*,†^*P* < 0.05, ^**,††^*P* < 0.01, and ^***,†††^*P* < 0.001 (^*^Wilcoxon rank-sum test, ^†^Wilcoxon signed-rank test).

**Supplementary figure 2. Gating strategy of mass cytometry and viSNE analysis**

(**a**) Representative gating strategy of singlets live CD45^+^CD3^+^CD4^+^ cells and (**b**) singlets live CD45^+^CD3^−^CD66a^−^CD56^−^CD19^−^CD161^−^ cells used in mass cytometric analyses. (**c**) Representative viSNE analysis CD163^+^ cells gated on live CD45^+^CD3^−^CD66a^−^CD56^−^CD19^−^CD161^−^ cells at 12 months. (**d**) The proportion of CD163^+^ cells gated on live CD45^+^CD3^−^CD66a^−^CD56^−^CD19^−^CD161^−^ cells. The open symbol represents responders, whereas the red filled symbol represents non-responders; ^†^*P* < 0.05 (^†^Wilcoxon signed-rank test). M, months.

**Supplementary figure 3. Induction of experimental cGN model, representative gating strategy, and dot plots of flow cytometry analysis.**

(**a**) Schematic for the modified accelerated cGN model. (**b**) Gating strategy of mouse kidney sample gated on singlets, FVD^−^CD45^+^CD4^+^CD8a^−^, to analyze IFNγ^+^, IL-17A^+^, and FoxP3^+^ cells. (**c**) Gating strategy of flow cytometry analysis on mouse kidney sample gated on singlets, FVD^−^CD45^+^, CD4^−^CD19^−^CD49b^−^Ly6G^−^ (lineage negative) cells to analyze the expression of CD206 and relative mRNA of CCL22/17.

**Supplementary figure 4. Analysis of *Il-6***^−/−^ **mice.**

(**a**) Kidney function was measured based on the levels of serum blood urea nitrogen (BUN) and creatinine and based on the ratio of urine albumin (UAlb)/urine creatinine (UCr) (n = 6). Glomerular crescent formation and interstitial inflammation were evaluated via renal pathological findings (n = 6). (**b**) Representative dot plots of flow cytometry analysis gated on CD45^+^CD4^+^FVD^−^ cells isolated from the kidney (n = 6). The proportion of IFNγ^+^, IL-17A^+^, and FoxP3^+^ cells per CD45^+^CD4^+^FVD^−^ cell in the kidney (n = 6). Symbols represent individual data points of mice representative of three independent experiments, and the horizontal lines indicate the mean ± SEM; ^*^*P* < 0.05 and ^**^*P* < 0.01 (Student’s *t*-test).

**Supplementary figure 5. Analysis of MR16-1 treatment at the early phase**

(**a**) Schematic for MR16-1 treatment regimen. Intravenous infusion of MR16-1 at an initial dose of 2 mg/mouse before being triggered by an GBM antibody injection and 0.5 mg/mouse thereafter. (**b**) Glomerular crescent formation and interstitial inflammation were evaluated via renal pathological findings. The kidney function was assessed by measuring serum blood urea nitrogen (BUN) and creatinine levels and by the ratio of urine albumin (UAlb)/urine creatinine (UCr) (n = 4 or 7). (**c**) Representative dot plots of flow cytometry analysis gated on CD45^+^CD4^+^FVD^−^ cells isolated from the kidney (n = 4 or 7). The proportion of IL-4^+^ cells and FoxP3^+^ cells per CD45^+^CD4^+^FVD^−^ cells in the kidney. (**d**) Relative mRNA levels of CCL22/17 normalized to GAPDH extracted from kidney tissue. Symbols represent individual data points of mice, and the data were pooled from two independent experiments. The horizontal lines indicate mean ± SEM; ^*^*P* < 0.05, ^**^*P* < 0.01, ^***^*P* < 0.001, and ^****^*P* < 0.0001 (Tukey–Kramer HSD test and Steel–Dwass test).

**Supplementary figure 6. Representative dot plots of flow cytometry analysis in *Cd3ε****^−/−^* **mice.**

Representative dot plots of flow cytometry analysis gated on FVD^−^CD45^+^CD3e^+^CD4^+^ cells isolated from the kidney, renal lymph nodes (LN), and mesenteric LN in *Cd3ε*^−/−^ mice.

**Supplementary figure 7. CCL17 had a lower migration ability than CCL22.**

(**a**) Schematic of the CCL17 treatment regimen, which was the same as that of Figure 4; 4 μg per mouse and 2 μg per kidney; procedure in detail is described in Supplementary Table S4. (b) Glomerular crescent formation and interstitial inflammation were evaluated in renal pathological observations. Kidney function was assessed by measuring serum blood urea nitrogen (BUN) and creatinine levels, and according to ratios of urine albumin (UAlb)/urine creatinine (UCre) (n = 3, 6, or 9). Symbols represent individual data points from mice that were representative of two independent experiments. (**c**) Representative dot plots from flow cytometry analyses gated on FVD−CD45^+^CD4^+^ cells that were isolated from kidneys; proportions of IFNγ^+^, IL-17A^+^, and CCR4^+^FoxP3^+^ cells per FVD^−^CD45^+^CD4^+^ cell in the kidney (n =3, 6, or 9). (**d**) *Ccl22/17* mRNA expression levels were normalized to those of *Gapdh* in kidney tissues in WT mice on days 0, 10, and 28*.* Symbols represent individual data points from mice in two representative, independent experiments, and horizontal lines indicate means ± standard errors of the mean (SEM); ^††^*P* < 0.01 vs. day 0 (Tukey–Kramer HSD test) and ^*^*P* < 0.05 (Student’s t-test). (**e**) The migration index was calculated as the number of cells migrating toward the concentration gradient of chemokines divided by the number of cells migrating toward medium only (n = 3). Symbols represent individual data points from wells that were representative of two independent experiments, and horizontal lines indicate mean ± SEM; ^*^P < 0.05 (Student’s *t*-test or Wilcoxon rank-sum test).

**Supplementary tables**

**Supplementary table 1. Baseline clinical characteristics and outcome of patients with MPA in TCZ monotherapy at 12 M.**

MPA patients newly diagnosed according to the Watts’ classification algorithm (TCZ group, n = 9), based on our previous reports and two additional patients. BVAS, Birmingham Vasculitis Activity Scores; Cr, serum creatinine; CR, complete remission; CRP, C-reactive protein; CPFE, combined pulmonary fibrosis and emphysema; D, dermal involvement; E, early systemic disease type; F, Focal type; G, gastric involvement; Ge, Generalized disease type; IGRA, interferon-gamma release assay; ILD, interstitial lung disease; IPMN, intraductal papillary mucinous neoplasm; M, Mixed type; MPO-ANCA, myeloperoxidase-antineutrophil cytoplasmic antibody; N, peripheral nerve; NSIP, non-specific interstitial pneumonia; P, pulmonary involvement; PMH, past medical history; R, renal involvement; S, Sclerotic type; UIP, usual interstitial pneumonia; UP, urine protein; VDI, vasculitis damage index.

**Supplementary table 2. Clinical characteristics of patients with MPA in corticosteroids at a single time of blood sampling.**

MPA patients who showed remission status with only low-dose corticosteroids (CSs) during conventional maintenance therapy and had at least one flare-up experience after the first induction (CS group, n = 6). BVAS, Birmingham Vasculitis Activity Score; C, cardiac involvement; CRP, C-reactive protein; CYC, cyclophosphamide; MPO-ANCA, myeloperoxidase-antineutrophil cytoplasmic antibody; N, peripheral nerve; P, pulmonary involvement; PSL, prednisolone; R, renal involvement; VDI, vasculitis damage index.

**Supplementary table 3. Clinical characteristics of healthy control individuals.**

**Supplementary table 4. List of products and *in vivo* experimental procedure.**

Refer to another file.
